# The Present Status of the Goitre Problem

**Published:** 1915-03

**Authors:** G. W. Cottis

**Affiliations:** Jamestown, N. Y.


### BUFFALO MEDICAL JOURNAL

Yearly Volume 70. MARCH, 1915. No. 8

### ORIGINAL ARTICLES

The right is reserved to decline papers not dealing with practical medical and surgical subjects, and such as might offend or fail to interest readers. Contributors are solely responsible for opinions, methods of expression and revision of proof.

### The Present Status of the Goitre Problem.

By G. W. COTTIS, M. D., F. A. C. S.

To an audience of surgeons, a textbook dissertation on goitre is neither of value nor of interest. On the other hand, a consideration of this subject as one of the great unsolved problems of medicine should be worth while. The very multiplicity of papers dealing with the many phases of the question renders it advisable to occasionally take an inventory, in order that we may pick out that which is of value to us as practitioners and students.

This problem is at about the same stage of solution as the cancer problem. In both we have the accumulated observations of centuries, the facts added to these observations by modern research and the still unknown facts necessary to a solution. My purpose is to arrange as many as possible of the known terms of the equation, in the hope that in the discussion some approximate value for X may be determined.

First it is necessary to keep in mind the facts established by many years of observation, because no solution can be accepted which is not in harmony with them. The more important of these facts may be briefly summarized as follows:

Goitre is of world-wide distribution as either an endemic, epidemic or sporadic disease. It was known in very early time as being endemic in regions where it still is endemic. Regions of endemic goitre are well circumscribed, and non-goitrous persons and lower animals moving into these areas are likely to contract goitre. Goitres thus acquired tend to disappear when the affected person or animal leaves the district. Furthermore, epidemics of goitre occur chiefly in goitrous regions and never spread to persons living near but not in the same area.

Drinking water has some causal relation to the disease. Goitre wells have been known for centuries, and the number of goitres in certain goitrous areas has been much reduced by the introduction of water from non-goitrous regions.

Altitude and latitude have no constant influence.

Congenital goitres are rare outside of goitrous regions. Acquired goitre occurs most frequently at about the age of puberty, the tendency diminishing after twenty.

Sex is a marked factor, the ratio of women to men being given as about eight to one. In Jamestown my own records show seven men to seventy women, a ratio of ten to one.

There is some connection between the thyroid and the sexual organs, the gland swelling very frequently at puberty, during pregnancy and at the menstrual period.

These are the more important facts established by the older investigators.

PATHOLOGY:

Much of our present confusion of ideas is due to the fact that the word ''goitre'' is used to describe an undetermined number of different diseases. Excluding syphilis, tuberculosis and malignant tumors, the following anatomic classification of goitre is the one most generally accepted:

1. Colloid goitre, which resembles a normal thyroid except for the increased amount of colloid distending the acini and causing pressure atrophy of the epithelium.
2. Adenomata, of which there are two varieties, the so-called fetal type composed of solid masses of cells, the acini having no lumina containing colloid, and the adult type in which the acini have lumina containing colloid.
3. Hyperplastic goitre, which is self descriptive.

To these we should, perhaps, add inflammatory swelling of the gland on account of its being a possible factor in the etiology of the other three types.

The relation of this anatomic classification to the classification based on symptoms is both interesting and important. The old clinical division into simple and exophthalmic goitre will no longer suffice.

Wilson found that of so-called simple goitres 45 per cent. were composed principally of adenomata and 44 per cent. consisted chiefly of colloid goitre, while only .6 per cent. showed hyperplasia. Of the cases classed as exophthalmic goitre 79 per cent. showed primary hyperplasia, 11 per cent. showed hyperplasia due to a secondary regeneration of an old colloid goitre, and 10 per cent. were adenomata, .9 of which were of fetal type.

Plummer's clinical classification is the most satisfactory one thus far suggested. He distinguishes four classes:

1. Non-hyperplastic non-toxic.
2. Non-hyperplastic toxic.
3. Hyperplastic toxic.
4. Hyperplastic non-toxic.

The last combination occurs so rarely that it probably represents errors in observation. Practically we meet only the first three types. The first two—the non-hyperplastic—include the colloid and adenomatous goitres, and of these 77 per cent. forming group one cause no constitutional symptoms, while the 23 per cent. composing group two produce toxic symptoms more or less closely resembling Graves disease, but never causing exophthalmos. Group three comprises all the cases of true exophthalmic goitre, only .8 per cent. of all hyperplastic goitres appearing to be non-toxic. It is this .8 per cent. which constitute group four.

Marine's views are directly opposed to those of the Mayo workers. He maintains that all forms of goitre are different stages of the same disease; that hyperplasia is always the first stage; that a colloid goitre is simply a cured hyperplastic goitre; and that adenomata never occur in an otherwise healthy thyroid. Furthermore, his experiments on goitrous puppies show that a true thyroid hyperplasia can be changed to a simple colloid gland by the administration of iodine in less than one week. He points out that the pathology of Graves disease is not confined to the thyroid, but that the lymphoid hyperplasia throughout the body is quite as distinctive and constant as is the changes in the thyroid.

The goitre he believes to be a part of the general reaction of the body to some profound constitutional disturbance, probably nutritional in character. He sums up his views on exophthalmic goitre by saying, "The essential physiological disturbance of the thyroid in exophthalmic goitre is insufficiency, its reaction compensatory, and its significance symptomatic."

Nobody so far as I know has tried to explain the lymphoid hyperplasia of toxic goitre. Most writers ignore it although Kocher emphasizes the importance of lymphocytosis both in diagnosis and prognosis. Many writers refer to the enlargement of the thymus and there are cases reported where thymectomy promptly cured the disease after thyroidectomy had failed. Every one who has studied the thyroid histologically has noticed the masses of lymphoid tissue between the acini in both hyperplastic and non-hyperplastic toxic goitres. In two cases of true exophthalmic goitre in young girls, I have seen the mononuclear leucocytes increase to 56.6 per cent. and 60 per cent. respectively, while in another case fifteen hours before death under medical treatment the lymphocytes were reduced to 10 per cent. showing the loss of compensatory reaction.

ETIOLOGY:

If we are still in doubt as to the ultimate nature of thyroid disease from the standpoint of pathological physiology, we are still more at sea regarding etiology.

McCarrison has demonstrated beyond a doubt that simple goitre is water borne, and his work is strongly suggestive of its causation by some intestinal organism.

Gaylord believes that his observation on endemic goitre in trout indicate an infectious origin and he produced goitre in dogs by feeding the sediment from the ponds containing the goitrous fish.

Bircher was able to induce goitre in rats by feeding them water from a goitrous region, but the same water when shipped to Berlin failed to produce the disease.

Recently many writers have adduced evidence pointing toward focal infection. For example, Lane claims to cure goitre by his operation of colectomy. Billings reports seven cases associated with tonsilitis or alveolar infections, in which cure or great improvement followed treatment of the infected tissues. A striking feature is that five of these seven cases were toxic goitres. He quotes Vincent as saying that thyroiditis occurred in 68 per cent. of cases of acute rheumatism.

Still more significant is Rosenau's report that by improved methods he has isolated a Gram-positive diplobacillus from the thyroid in twenty-five out of thirty-two operative cases of goitre ("Mostly exophthalmic") and from the blood on two occasions in a severe case of exophthalmic goitre, while his results were negative in six out of seven dogs with normal thyroid. He also grew an anaerobic hemolytic staphylococcus from most of the goitres examined both in dogs and men.

I have had two cases of acute swelling of the thyroid with severe exophthalmic goitre occurring after a week or two of some grippy infection. In one the neck was swollen, red and tender, the patient stuporous at times with some delirium, a pulse of 160 and high fever. I operated, expecting to find pus but removed part of the enlarged isthmus without either pus or hemorrhage from the cut service. Section showed interstitial thyroiditis. The patient promptly recovered. The other case appeared to be a typical acute exophthalmic goitre with all the classical symptoms including goitre and exophthalmos developing within ten days. Ligation was followed by severe reaction including delirium. Thyroidectomy was necessary one year later for persistent tachycardia and nervousness.

FUNCTION:

Present evidence indicates four well defined functions of the thyroid although the exact mechanism of none has been thoroughly worked out.

1. Its relation to the growth of the body is well recognized. It is necessary only to mention the imbecilic cretin dwarf produced by the hypothyroidism of congenital goitre.

2. Closely related to its influence on the growth of the body is its power of increasing oxidation and nitrogenous metabolism. Beebe says that by the administration of thyroid to a cretin or a patient with myxoedema it is possible to increase the absorption of oxygen from 20 to 75 per cent. Masoin found that the relative quantity of oxyhemoglobin was diminished gradually as the postoperative phenomena of thyroidectomy progressed. This is of especial interest in relation to pregnancy. The work of Williams shows that the nitrogen partition of the urine is the most positive index to the toxemias of pregnancy. He states that the normal ammonia co-efficient in pregnancy is four to five per cent., while in toxic vomiting it may rise to 10, 20, 30, or even 40 per cent. Now, MacCallum and Voetglin have proved that thyroidectomy causes a greatly increased ammonia content in the blood, and Carlson has shown a marked depression of the ammonia destroying power of the liver after complete removal of the thyroid. This means that the liver has an ammonia destroying or converting power; when it fails in this function ammonia appears in the urine and also in the blood. This failure regularly occurs in the toxemia of pregnancy and also after thyroidectomy. Finally, Ward has recently published clinical corroboration in the form of several case reports of severe toxemia in pregnancy with corresponding high nitrogen output, in which thyroid administration produced diminution in the nitrogen and a simultaneous clearing up of the toxic symptoms. I have read the statement but cannot find the authority for it, that the thyroid enlargement of pregnancy is never found in toxic cases.

3. Thyroid products have something to do with immunity to infections. Lack of thyroid secretion always means diminished resistance. Sajous maintains that iodothyrin actually is identical with the hypothetical opsonins of Wright. Many workers have shown that the germicidal alexins and hemolysins of the blood are diminished by thyroidectomy and increased by thyroid administration. Conversely goitre has been produced by the injection of bacterial toxins into the blood.

4. Finally we have the function of activating the other ductless glands, especially the adrenals and the pituitary. Hoskins, Kraus and others have shown that while normal blood has no mydriatic power the blood of hyperthyroidism has, and this same mydriatic activity, due to adrenalin, is produced in the serum by the administration of thyroid and the mydriatic power varies directly with the dose administered. This has a direct bearing on Crile's kinetic theory of exophthalmic goitre but time will not permit a discussion of it here.

It is not my purpose to say anything about treatment although that phase of the subject is not without interest. I believe it is more important to take the time in trying to correlate our knowledge of the real nature of thyroid disease. Let me suggest a few points of convergence for the various theories and facts which I have mentioned.

Evidence is rapidly accumulating that at least some types of goitres are caused by infections in other parts of the body. Most of those cited are toxic and therefore probably hyperplastic. Marine tells us that colloid goitres are always end results of hyperplasia. It is possible that a mild thyroiditis is present more often than we know in such diseases as scarlatina, tonsilitis and other diseases of childhood, and that juvenile goitres start from these infections.

Gaylord's work in producing goitre in dogs by feeding them sediment from ponds containing goitrous fish and McCarrison's prevention of goitre by boiling goitrous water or treating it with chlorine, suggest the probability that the so-called non-toxic goitres represent a slow reaction to chronic infections, as the toxic ones do to acute infections and that all of them are really protective in their nature.

Are thyroid adenomata, comprising nearly one-half of all chronic goitres, a reaction to stimuli furnished by localization of infection in the gland just as vegetal tumors result from specific bacterial infections?

Are not the goitres of pregnancy merely a response to the demand for neutralization of toxin and hence purely physiological and protective?

CONCLUSIONS:

There is strong evidence of the truth of each of the following statements:

1. Hyperplasia of the thyroid is the direct cause of the symptoms of exophthalmic goitre. Lymphoid hyperplasia is equally as constant in the pathology of the disease, but its significance is not known. It is a distinct disease having no direct relationship to endemic goitre.

2. Some acute toxic goitres result directly, from stimulation of the thyroid by direct infection of the gland or by toxins from more remote infections. Others represent a reaction to a severe strain, physical, nervous or mental, necessitating an increased supply of thyroglobulin.

3. Chronic, non-toxic goitre is sometimes the end result of an acute toxic goitre; sometimes, and probably always in endemic goitre, the consequence of drinking bacterially contaminated water; and frequently it is merely the persistence of physiologic hypertrophy such as that which occurs in pregnancy.

If these statements prove to be true, it is evident that we are dealing with end results of several very different processes. It therefore follows that our treatment must be revised to attack the primary disease, instead of the final product of widely divergent causal conditions.

Jamestown, N. Y.

---

**Determination of Sugar in Blood.** Victor C. Myers and Cameron V. Bailey, Post-Grad., January, 1915. About 5 c. c. of blood are drawn into a tube containing 3 drops of 20% potassium oxalate to prevent clotting. Or, 2 c. c. are drawn through a hypodermic needle directly into an ostwald pipette containing a little potassium oxalate in the tip. 2 c. c. of blood plus the ovalate, are, at any rate discharged from an Ostwald pipette into a test tube, the adherent blood being saved by washing the pipette out with 8 c. c. of water, thus diluting the blood to 20% and securing thorough laking of the corpuscles. 0.20 of picric acid is added to precipitate proteid, the tube is left for several minutes and thoroughly stirred. With or without centrifugalizing, the mixture is filtered through a small thick paper, 7 c. m. in diameter. 3 c. c. of filtrate are pipetted into a tall narrow graduated test tube and 1 c. c. of 10% sodium carbonate is added. On standing in a beaker of boiling water, this develops the characteristic black-red color reaction of sugar. The mixture is further diluted with distilled water, 10 to 20 c. c. and read by a Hellige colorimeter, with a wedge of standard picramic acid solution. This solution consists of 0.1 gram picramic acid, 0.2 grams anhydrous sodium carbonate, dissolved at first in 30 c. c. of warm distilled water and finally made up to 1 liter. A table is given for more or less direct calculation of the sugar content according to the degree of dilution.

---

**Typhoid Following Anti-Typhoid Inoculation.** E. A. Bourke and S. Rowland, B. M. J., January 9, 1915, report the case of a soldier inoculated in March, 1914, and again November 10, three days before admission to hospital for what proved to be typhoid, death occurring from heart failure, December 24. (Note: It is highly important that all cases of this kind be reported promptly. It is quite generally held that any immunizing agent administered at the outset of the corresponding affection tends to increase the seriousness of the case. The present case even suggests the possibility of viable bacilli in the vaccine.)

